# Supplementary material for: Multivalent binding kinetics resolved by fluorescence proximity sensing
Source: Commun Biol. 2022 Oct 7;5:1070. doi: 10.1038/s42003-022-03997-3 (PMC9546861; doi:10.1038/s42003-022-03997-3)
Supplement: Supplementary file 2 — Supplemental Material [file 42003_2022_3997_MOESM2_ESM.pdf]

Supplementary Information

Fluorescence Proximity Sensing for Real Time Affinity Determination of Multivalent Peptide-Protein Interactions

Clemens Schulte[1]; Alice Soldà[2]; Sebastian Spänig[3], Nathan Adams[4], Ivana Bekić[4], Werner Streicher[4]; Dominik Heider[3], Ralf Strasser[2]; Hans Michael Maric[1]*

*Correspondence to hans.maric@virchow.uni-wuerzburg.de

[1]Rudolf Virchow Center; Center for Integrative and Translational Bioimaging; University of Wuerzburg; Josef-Schneider-Str. 2, Germany, 97080 Wuerzburg, Germany

[2]Dynamic Biosensors GmbH Germany, Lochhamer Strasse 15, 82152 Martinsried/Planegg, Germany

[3]Department of Bioinformatics, Faculty of Mathematics and Computer Science, Philipps-University of Marburg, Marburg, Germany

[4]Nanotemper Technologies GmbH, Flößergasse 4, 81369 Munich, Germany

Supplementary Figures

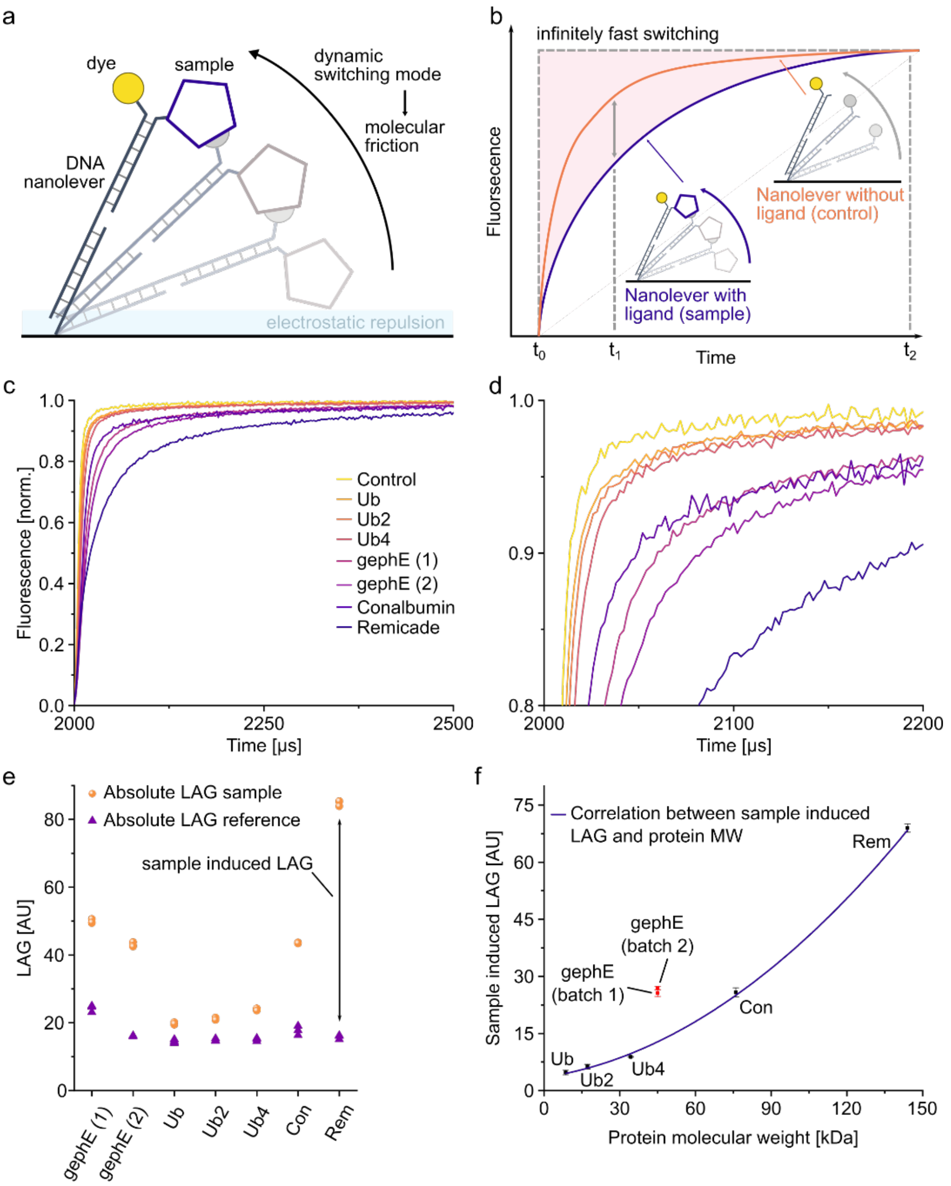

Supplementary Figure 1: switchSENSE relative size analysis – Dynamic Mode and Lag Value of GephE. (a) Theoretical explanation of the high frequency dynamic electrical switching mode, which probes the hydrodynamic radius (as a function of the friction) of analyte molecules and serves to determine the size and shape of biomolecules. The hydrodynamic radius of the ligand/sample (or hydrodynamic diameter) adds additional drag to the nanolevers when these are pushed and pulled through solution. Thus, the larger the ligand/sample, the slower the motion/switching speed. Therefore, the hydrodynamic radius of the conjugated protein (gephE) can be estimated by comparing the time-resolved fluorescence motion curve of gephE with switching curves of other proteins of know weight and size. (b) Theoretical explanation of the dynamic lag value. At a given time, the DNA nanolever with the ligand (sample) moves a shorter distance than the control DNA nanolever (control). It lags in distance and fluorescence behind the control, due to additional friction created by the ligand. The absolute dynamic lag corresponds to the area between the theoretical zero-drag nanolever (dotted grey line) and the control (blue line) or the sample curve (pink line), respectively. In accordance with increasing hydrodynamic friction, small ligands correspond to fast switching and large ligands to slower switching (*r* ~ dynamic lag). The sample induce-lag is the area between the control and the sample curve, i.e. the difference between the curve integrals. (c) fluorescence motion curves of all measured proteins (including two independently functionalized batches of gephE – (1) and (2)) and the respective control with an empty ligand strand. (d) zoomed-in version of panel (c). (e) Absolute LAG values of each protein and the respective control yield the sample induced LAG value. Individual values represented, n=3 (f) Sample induced LAG values of each measured protein are plotted against the respective molecular weight. Note that the sample induced LAG values measured for gephE (red) do not correlate with the rest of the LAG values that were measured for monomeric, globular proteins. This suggests that gephE is dimeric in the immobilized form. Values are presented as mean ± SD.

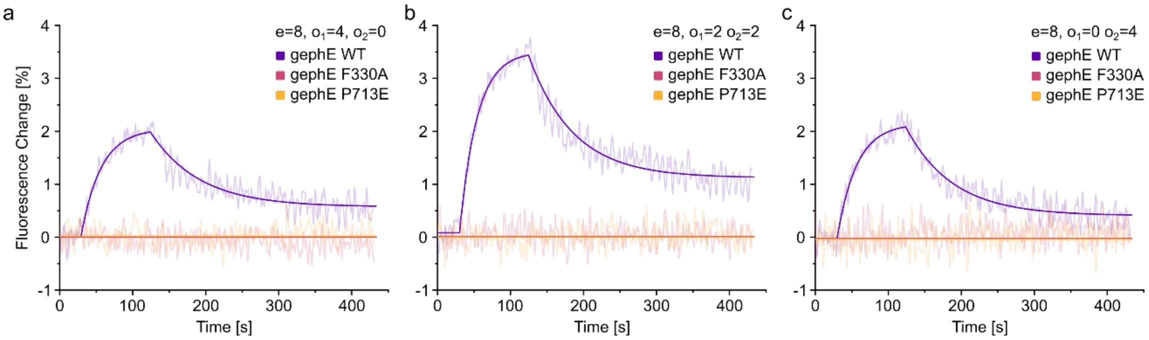

Supplementary Figure 2: Control experiment with two non-binding gephE point variants. Binding of three tetrameric peptides with varying architecture (a) e=8, o_1=4, o_2=0, (b) e=8, o_1=4, o_2=0, (c) e=8, o_1=4, o_2=0 to gephE wildtype and two non-binding point variants (P713E, F330A, first described in (Kim et al., 2006)) was evaluated in FPS. Note that neither one of the point variants exhibit binding to the tetrameric peptides in contrast to wildtype gephE.

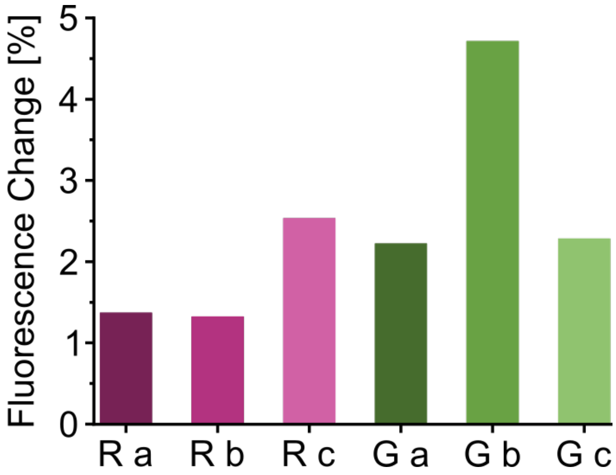

Supplementary Figure 3: Systematic dye scouting with red (R) and green (G) dyes revealed that green dye b (Gb, Dynamic Biosensors GmbH, DE) provided the highest fluorescence signal amplitude among all tested dyes. Signal amplitudes were determined in measurements with a purified, dimeric geph-binding peptide ((YSIVGSYPR)2pegKC) at a concentration of 0.1 µM.

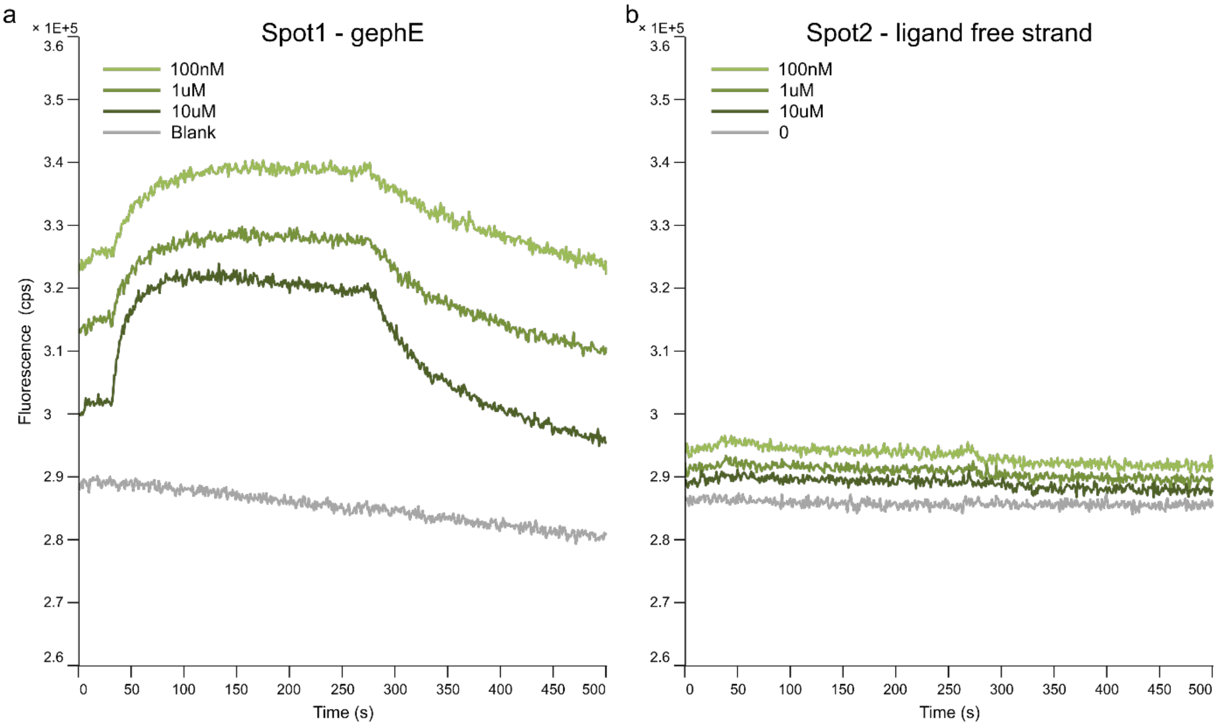

Supplementary Figure 4: Control conditions in FPS measurements. To account for a potential influence of the peptide on the fluorophore, a condition without immobilized ligand (gephE, panel (a)) is measured in parallel on spot 2 of the measurement chip (b) for each peptide concentration. In addition, a condition without peptide is used to monitor changes in baseline fluorescence.

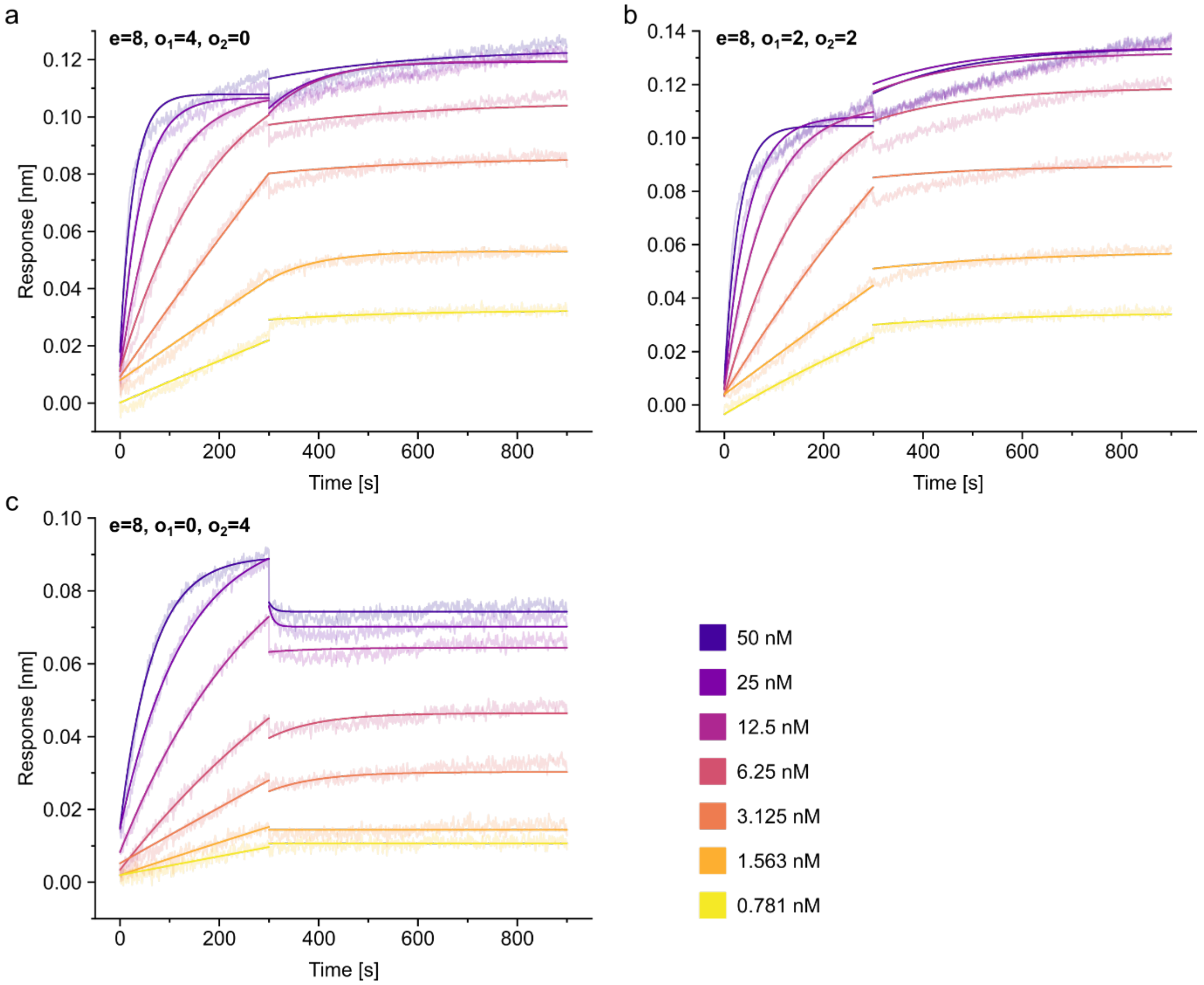

Supplementary Figure 5: BLI measurements of purified tetrameric peptides with 8mer epitopes and varying linker architecture. Tetrameric peptides with varying architecture (a-c) were subjected to BLI measurements at the indicated concentrations. Note that no off-rate could be determined for any of the tested tetramers.

65

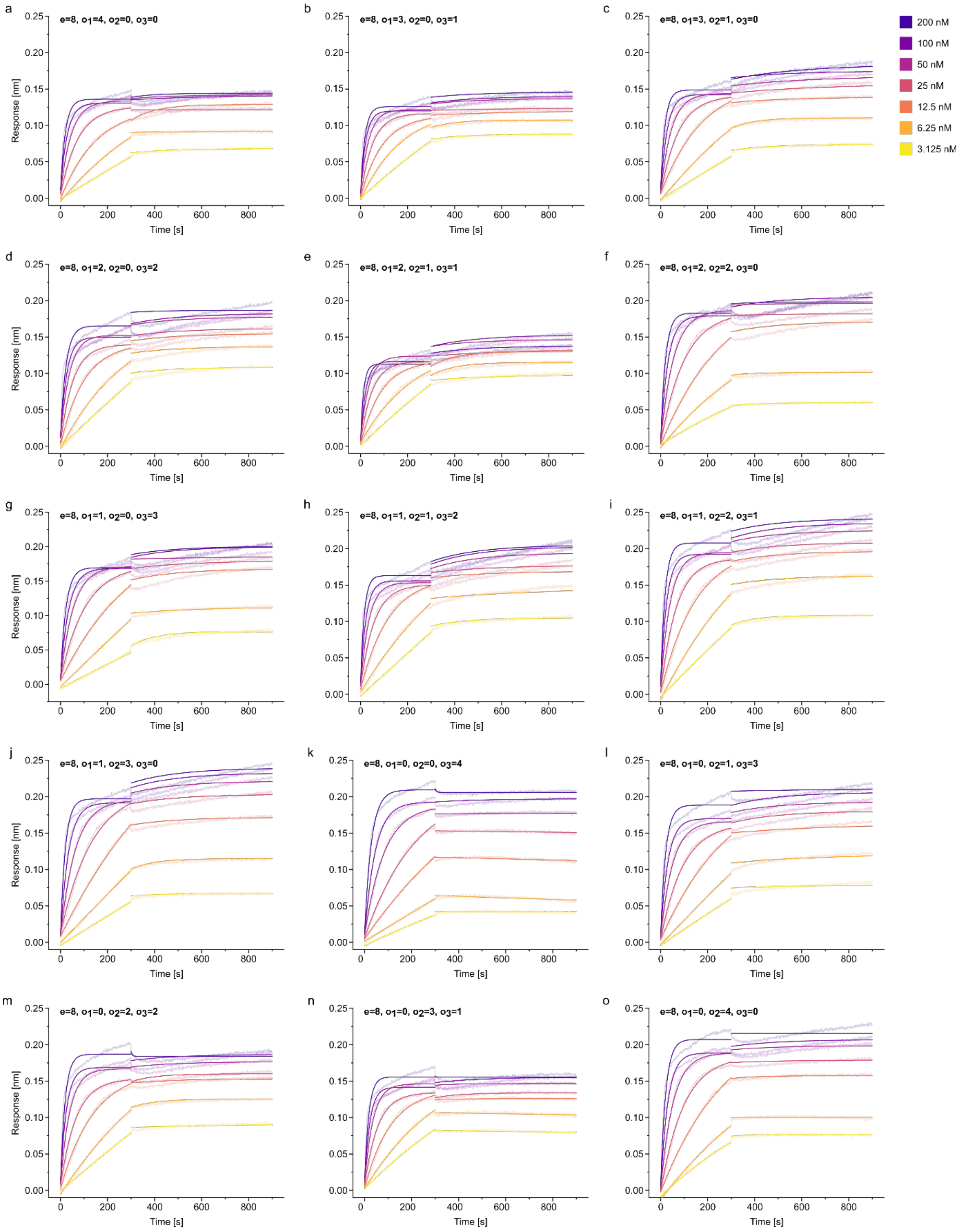

Supplementary Figure 6: BLI measurements of octameric peptides with 8mer epitopes and varying linker architecture. Octameric peptides with varying architecture (a-o) were subjected to BLI measurements at the indicated concentrations. Note that no off-rate could be determined for any of the tested octamers.

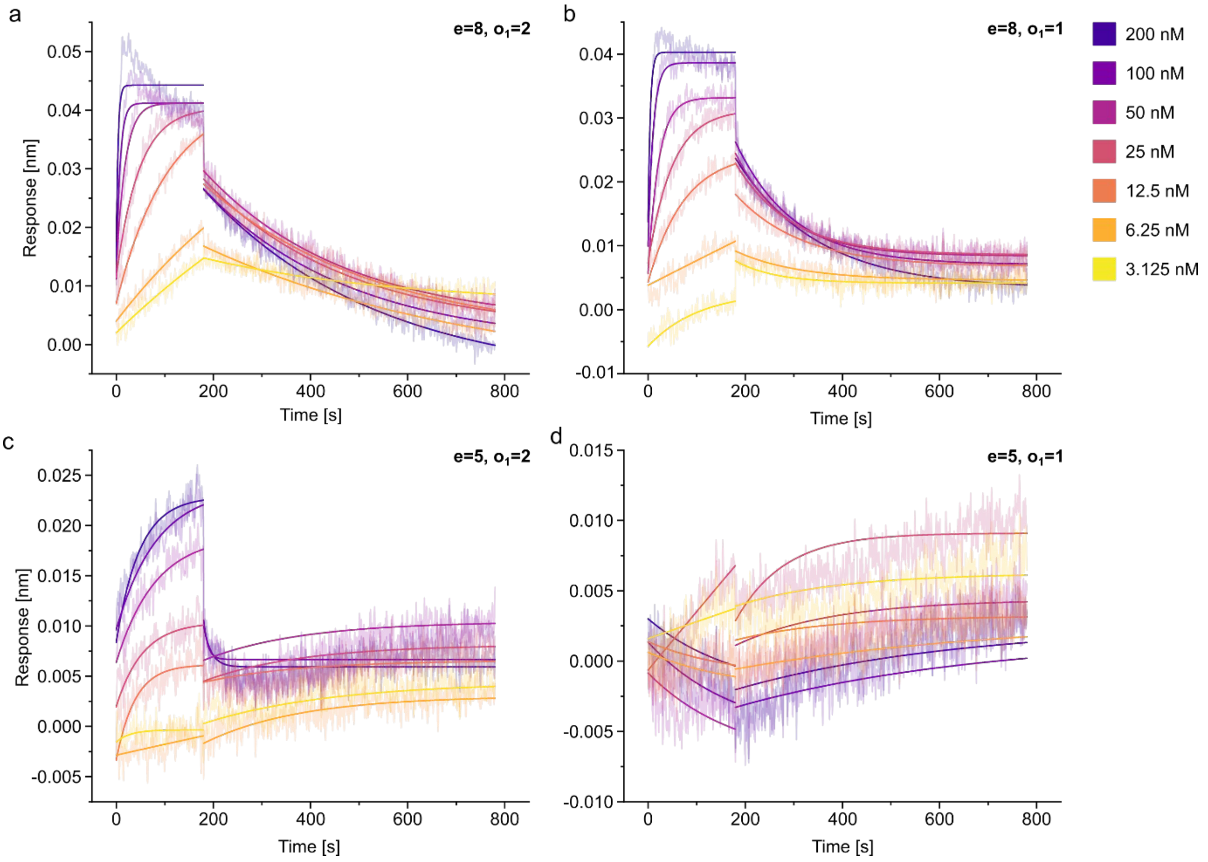

Supplementary Figure 7: BLI measurements of purified dimeric peptides with varying epitope and linker length. Dimeric peptides with varying epitope and linker length (a-d) were subjected to BLI measurements at the indicated concentrations. Note that for the smallest peptide tested (d), due to poor signal-to-noise ratio, no on- or off-rate could be determined.

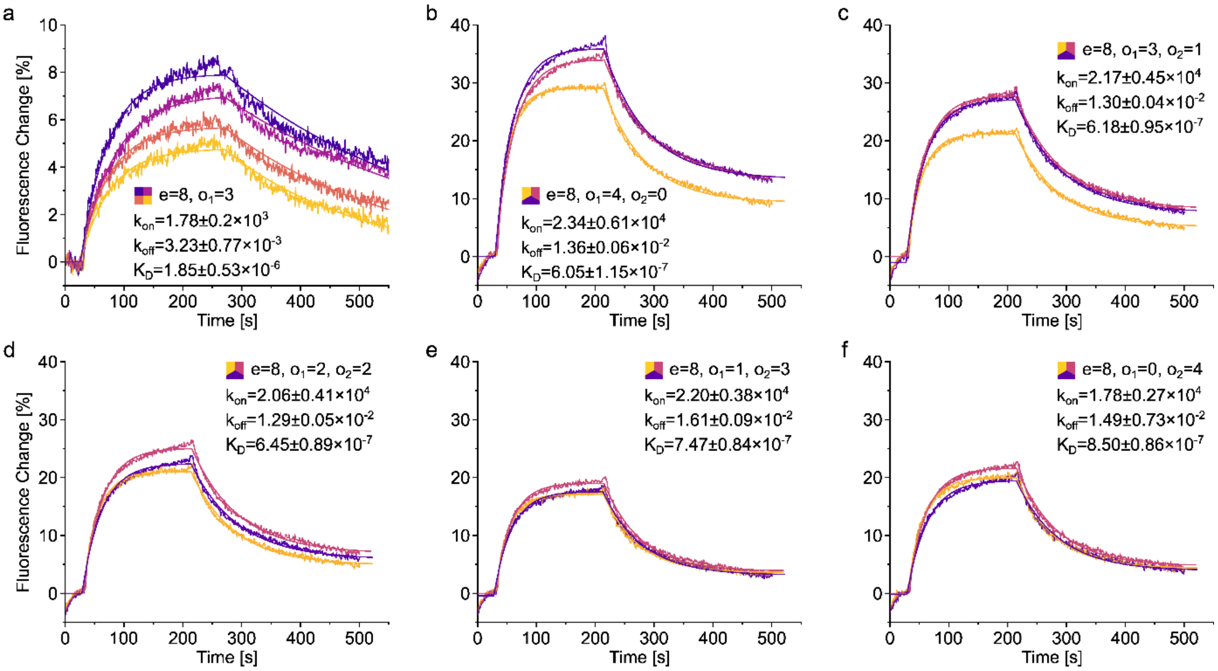

Supplementary Figure 8: High intra-synthesis reproducibility of k_{on}, k_{off}, and K_D values of unmodified peptides determined in FPS. To determine the intra-synthesis reproducibility of our assay setup, selected independently synthesized dimeric (a-b) and tetrameric (c-f) compounds were measured at a concentration of 1 µM. Dynamic K_D values and the corresponding k_{on} and k_{off} values are represented as mean with the corresponding

standard deviations. The dimeric peptide was synthesized and measured as n=4, the tetrameric compounds as n=3.

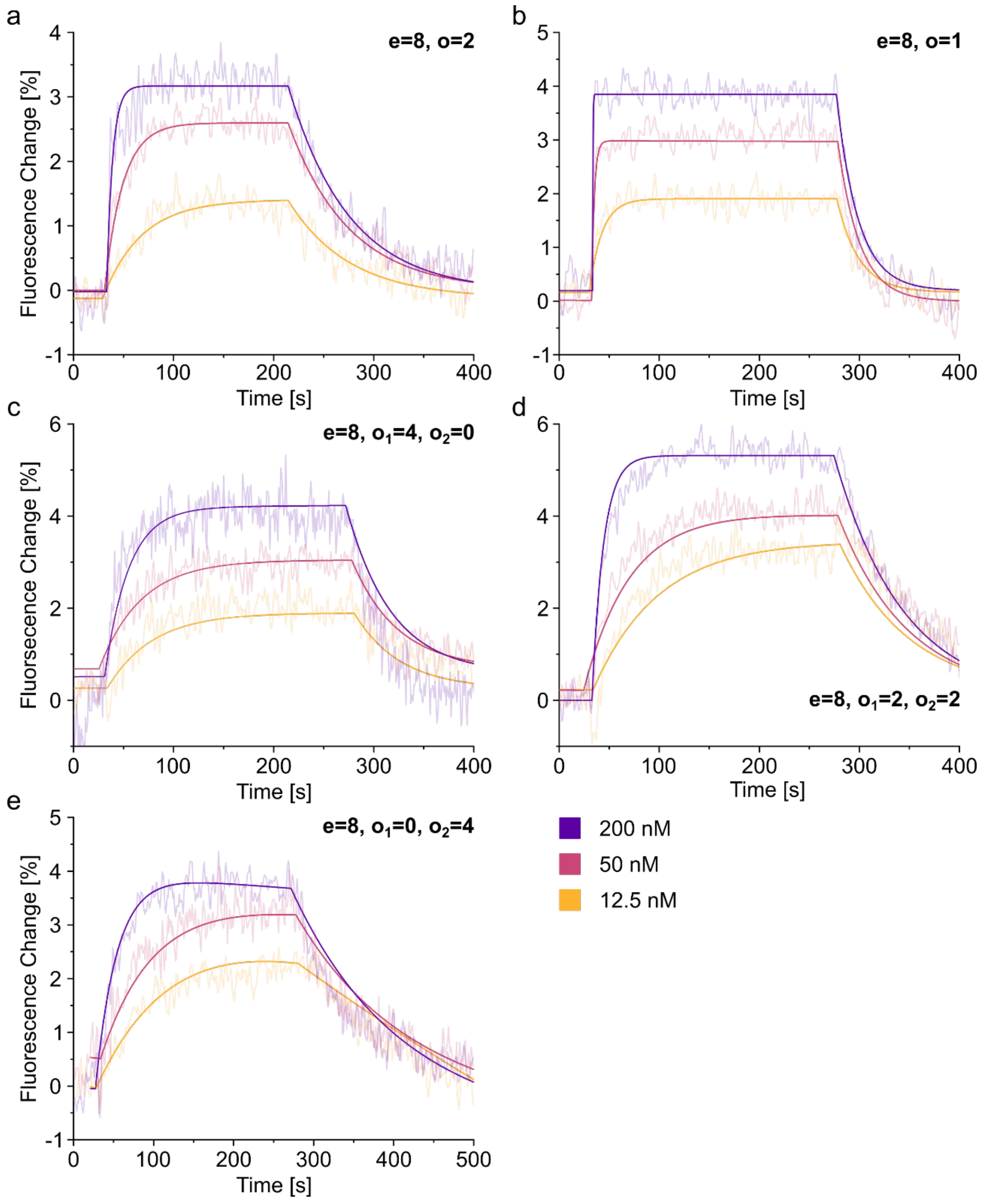

Supplementary Figure 9: Affinity determination of purified dimeric and tetrameric peptides in FPS. Dimeric (a-b) and tetrameric (c-e) compounds were subjected to FPS measurements at the indicated concentrations in the nanomolar range.

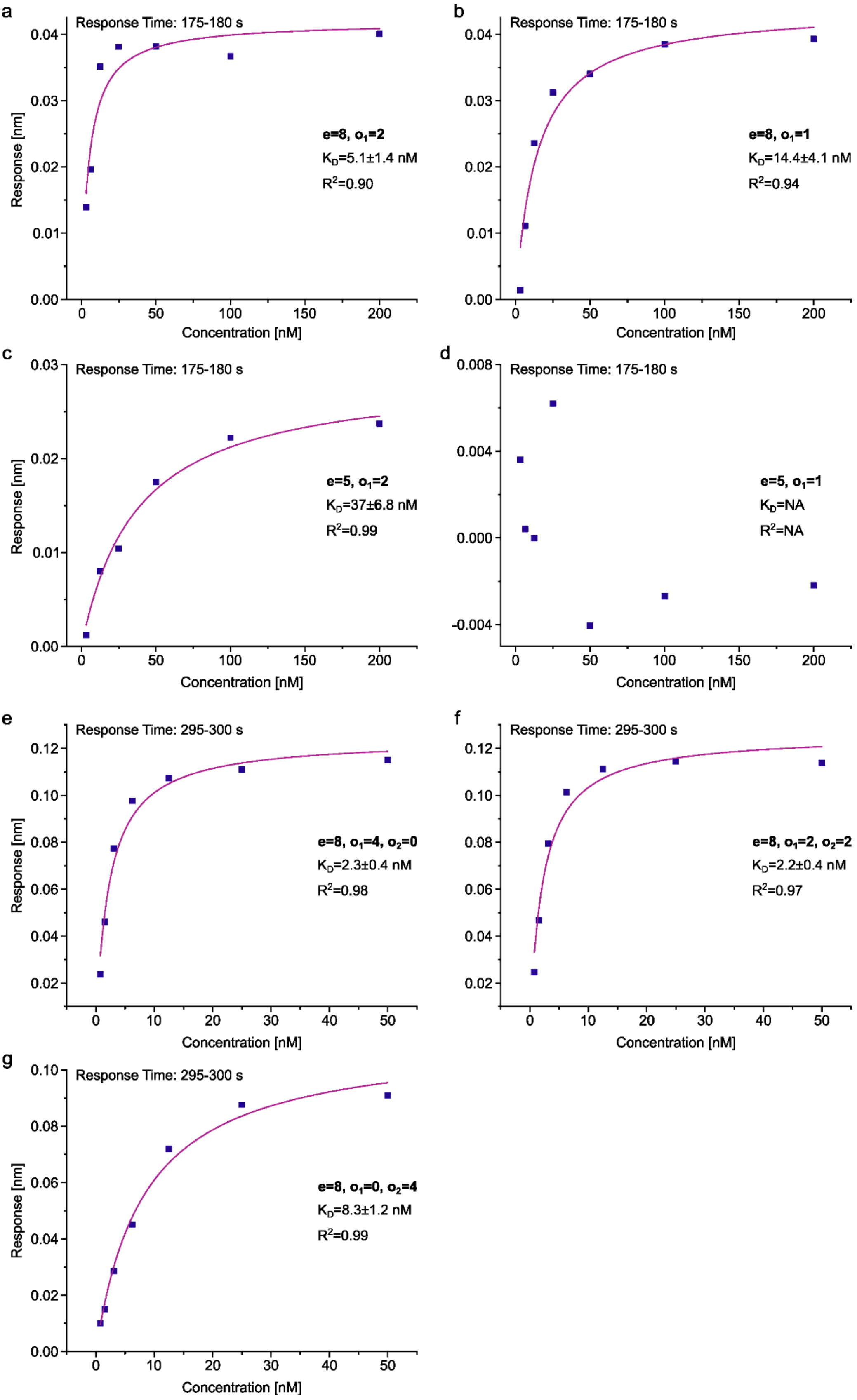

90 **Supplementary Figure 10: Affinity determination of purified dimeric and tetrameric peptides in BLI.** Steady-state responses from BLI sensograms (see supplementary figure 2 and 4) of the indicated dimeric (a-d) and tetrameric (e-g) peptides were plotted against the corresponding concentration and fit to determine the apparent steady state K_D values.

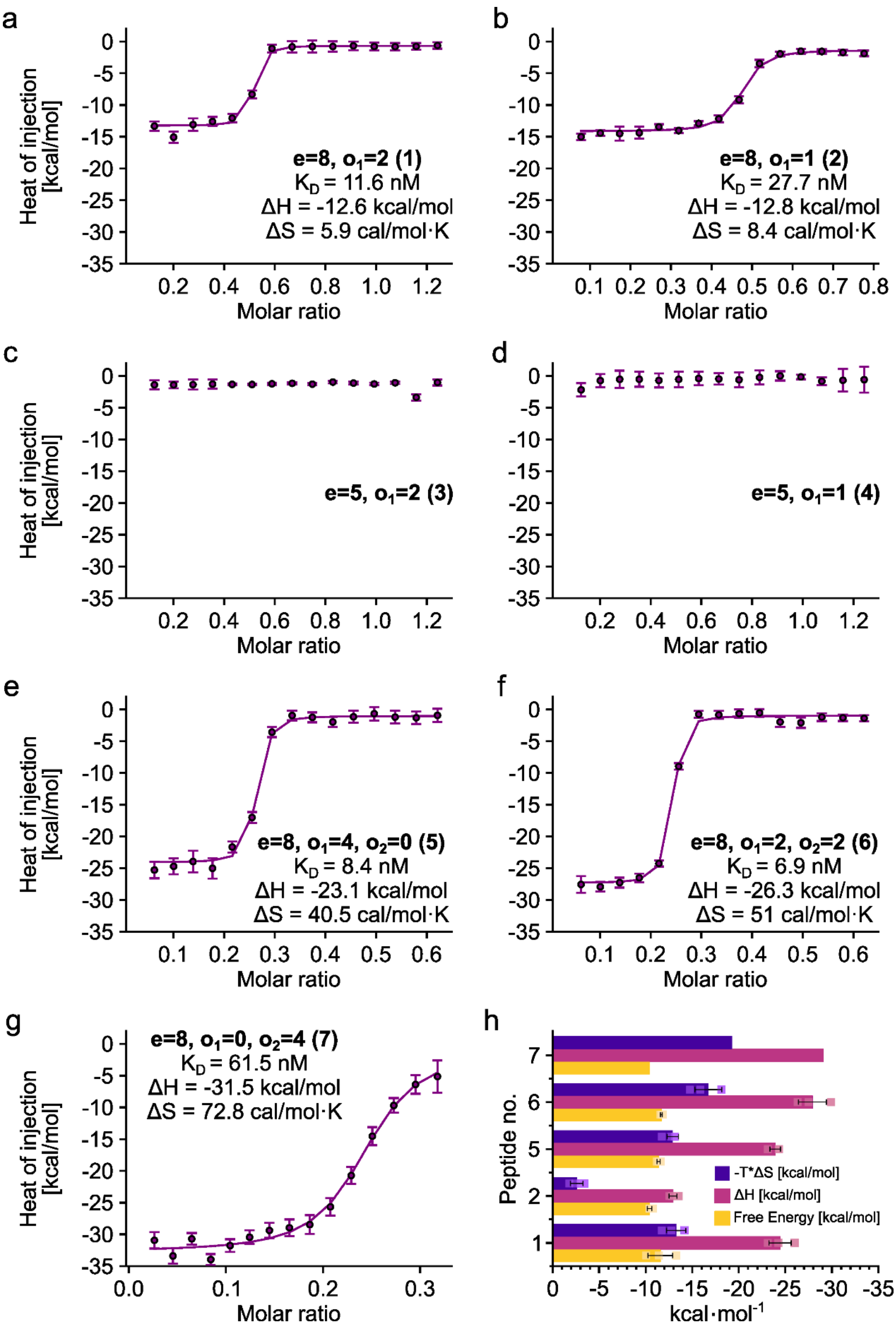

Supplementary Figure 11: representative binding isotherms of GephE titrated with purified dimeric and tetrameric peptides. (a-d) Integrated molar heat release obtained from titrating recombinant GephE with different dimeric peptides using the ITC method. (e-g) Integrated molar heat release obtained from titrating recombinant GephE with different tetrameric peptides using the ITC method. Error bars represent integration uncertainty and are added automatically in NITPIC. (h) Different contributions of the binding enthalpy and entropy to the overall free energy. Enthalpy, entropy and free energy bar graphs are averages and the corresponding standard deviations of three ITC measurements for (a)-(f) and the mean of two ITC measurements for (g).

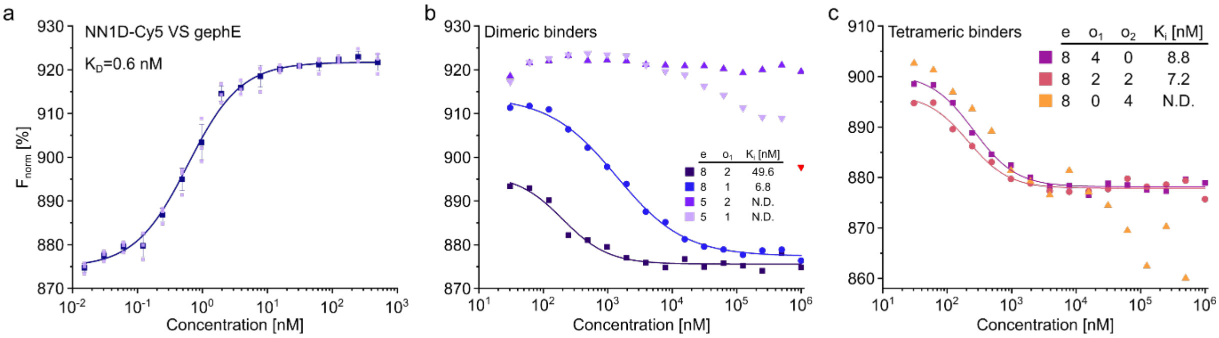

Supplementary Figure 12: Quasi label-free affinity determination of purified dimeric and tetrameric peptides in Dianthus. (a) Titration of gephE against a high affinity geph binding peptide with Cy5 (NN1D-Cy5) as a fluorescent tracer. Data are presented as n=3 ± SD. (b) Displacement assay with dimeric peptides with varying epitope length and linker architecture against a gephE – NN1D-Cy5 complex. (c) Displacement assay with tetrameric peptides with varying linker architecture against a gephE – NN1D-Cy5 complex. Values are presented as mean of n=2.

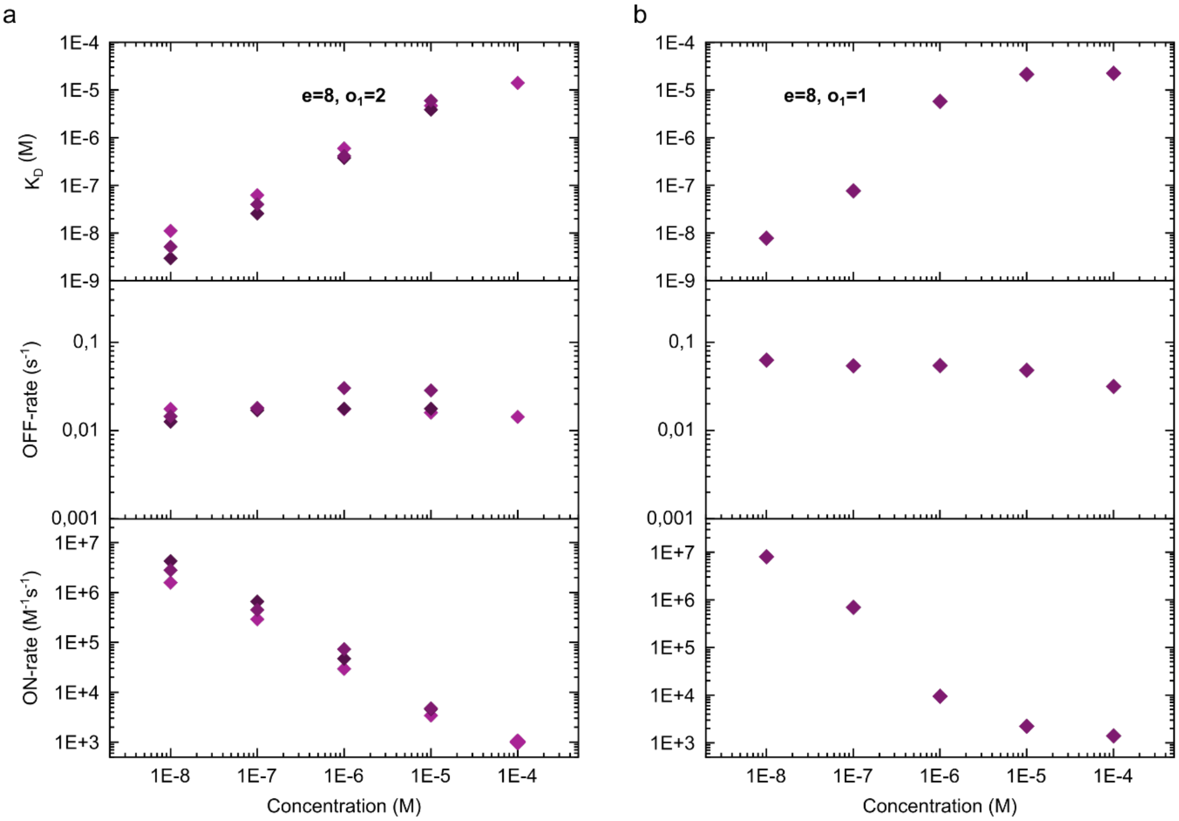

Supplementary Figure 13: Determined on-rates are dependent on the peptide concentration in FPS. Obtained on-, off-rates and K_D values at varying peptide concentrations of two dimeric peptides with an 8mer binding epitope (e=8) and a linker length two (a) or one (b) PEG building blocks.

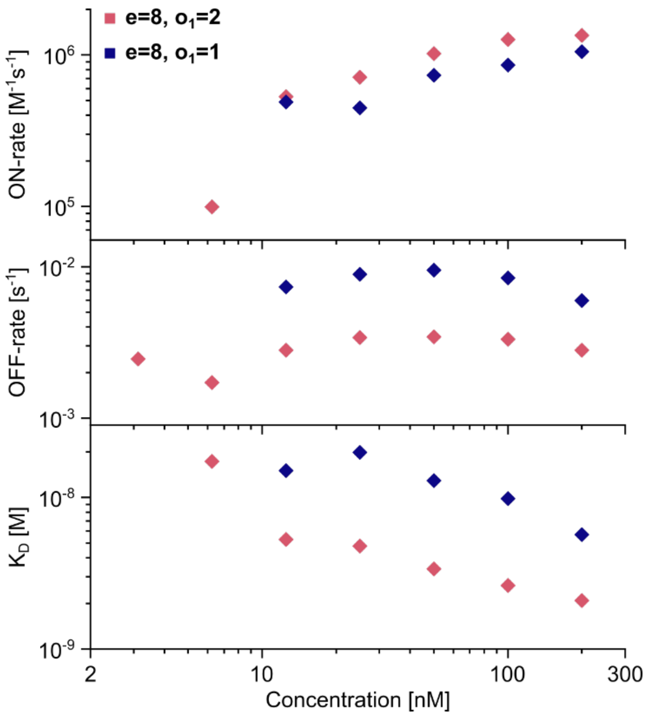

Supplementary Figure 14: Determined on-rates are dependent on peptide concentration in BLI. Obtained on-, off-rates and K_D values at varying peptide concentrations of two dimeric peptides with an 8mer binding epitope (e=8) and a linker length two (red) or one (blue) PEG building blocks.

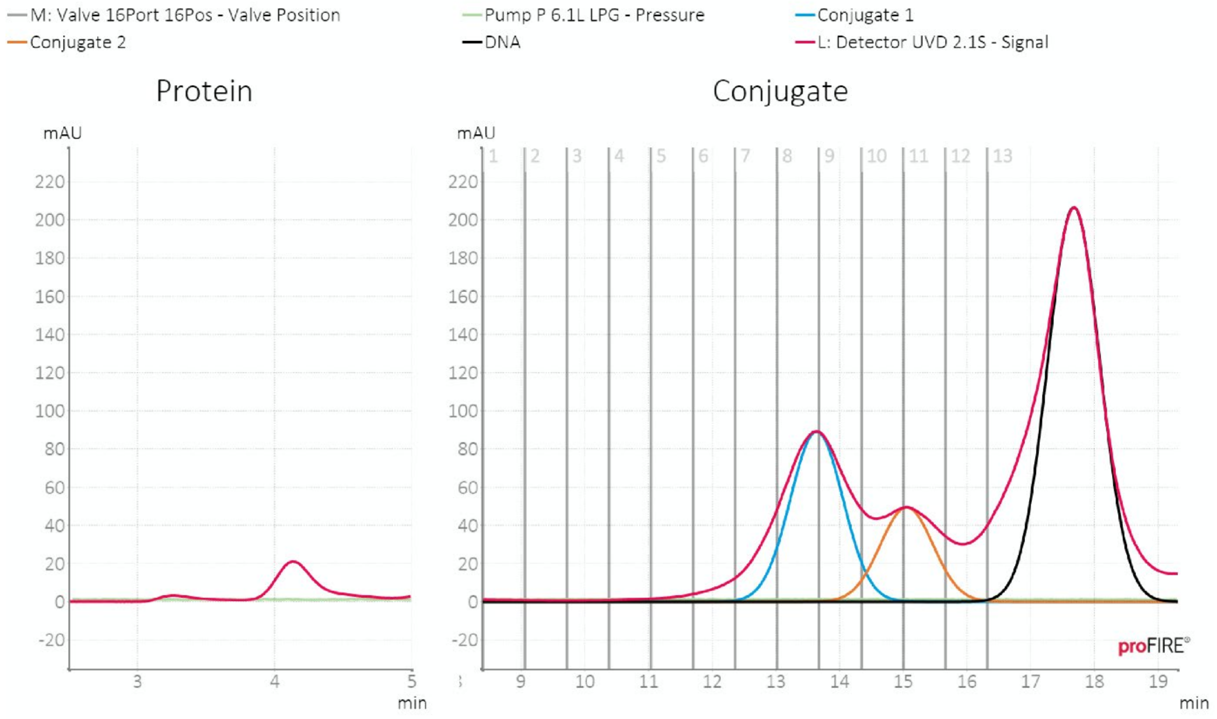

Supplementary Figure 15: proFIRE® purification chromatogram of GephE conjugated to a 48mer oligonucleotide. Conjugate peak 1 (blue line) shows a single population of a 48mer labelled protein with a 1:1 ratio. Conjugate peak 2 (orange line) shows a higher molecular weight, assuming a 2:1 ratio. The third peak (black line) is the excess of free DNA. Only peak 1 was used for the peptide screening.

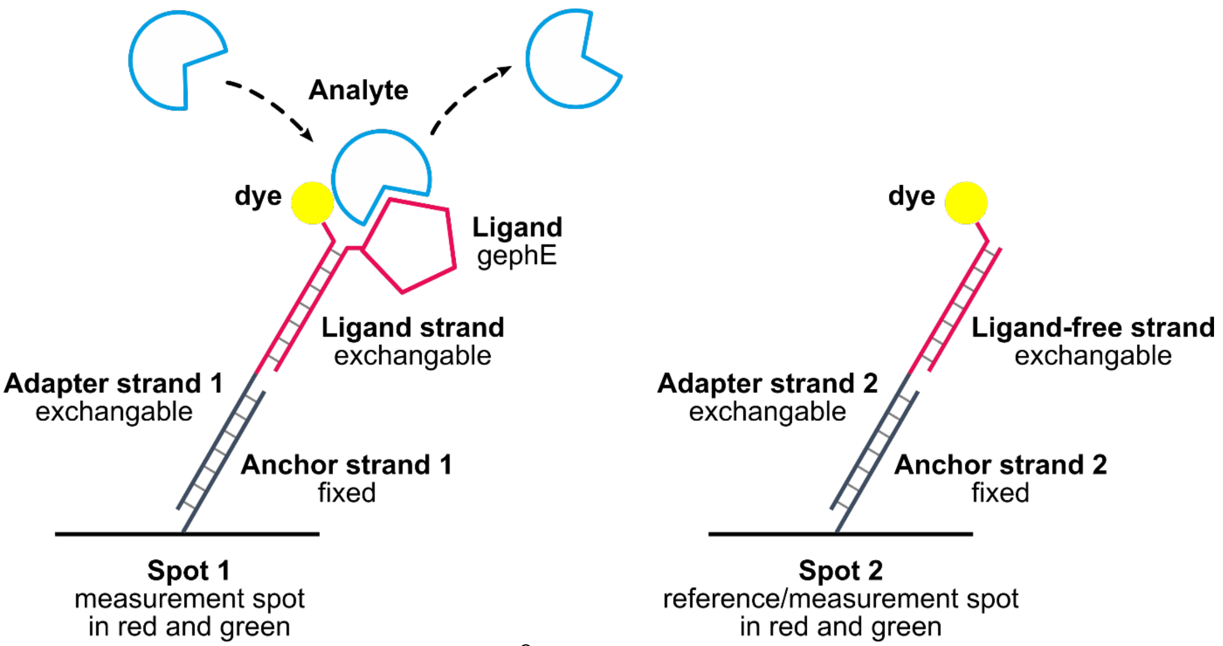

Supplementary Figure 16: Layout of the heliX® adapter biochip.

130

Supplementary Tables

Supplementary Table 1: Complete overview of dimeric, tetrameric and octameric peptides measured in FPS. Peptide sequences are given alongside the on-, off-rate and K_D value and corresponding STDEV obtained at peptide concentrations of 1 µM.

Peptide	Sequence	kon (M-1s-1)	STDEV	koff (s-1)	STDEV	KD (M)	STDEV	Association Level
GlyRβ_DIM_008_8_3P_WT001	FSIVGSLPOOOJ	1.63E+03	6.00E+01	4.42E-03	1.00E-04	2.71E-06	1.20E-07	4.43
GlyRβ_DIM_008_8_3P_WT002	FSIVGSLPOOOJ	1.91E+03	6.00E+01	3.39E-03	7.00E-05	1.77E-06	7.00E-08	5.49
GlyRβ_DIM_008_8_3P_WT004	FSIVGSLPOOOJ	1.54E+03	5.00E+01	2.51E-03	5.00E-05	1.63E-06	6.00E-08	6.85
GlyRβ_DIM_008_8_3P_WT005	FSIVGSLPOOOJ	2.05E+03	6.00E+01	2.59E-03	5.00E-05	1.27E-06	5.00E-08	8.04
GlyRβ_DIM_001	SDLRSNDFSIOOOJ	0.00E+00	0.00E+00	0.00E+00	0.00E+00	0.00E+00	0.00E+00	0.00E+00
GlyRβ_DIM_002	DLRSNDFSIVOOOJ	3.59E+03	5.10E+02	1.10E-02	9.00E-04	3.07E-06	5.00E-07	1
GlyRβ_DIM_003	LRSNDFSIVGOOOJ	0.00E+00	0.00E+00	0.00E+00	0.00E+00	0.00E+00	0.00E+00	0.00E+00
GlyRβ_DIM_004	RSNDFSIVGSOOOJ	0.00E+00	0.00E+00	0.00E+00	0.00E+00	0.00E+00	0.00E+00	0.00E+00
GlyRβ_DIM_005*	SNDFSIVGSLOOOJ	1.02E+04	3.10E+03	3.06E-02	3.60E-03	3.01E-06	9.90E-07	0.109
GlyRβ_DIM_006	NDFSIVGSLPOOOJ	6.35E+03	1.27E+03	1.98E-02	1.80E-03	3.12E-06	6.90E-07	1.04
GlyRβ_DIM_007	DFSIVGSLPROOOJ	5.15E+03	9.30E+02	1.39E-02	1.30E-03	2.71E-06	5.50E-07	1.11
GlyRβ_DIM_008	FSIVGSLPRDOOOJ	2.04E+03	2.20E+02	1.01E-02	6.00E-04	4.97E-06	6.00E-07	2.49
GlyRβ_DIM_009	SIVGSLPRDFOOOJ	0.00E+00	0.00E+00	0.00E+00	0.00E+00	0.00E+00	0.00E+00	0.00E+00
GlyRβ_DIM_010	IVGSLPRDFEOOOJ	0.00E+00	0.00E+00	0.00E+00	0.00E+00	0.00E+00	0.00E+00	0.00E+00
GlyRβ_DIM_008_10_3P	FSIVGSLPRDOOOJ	3.30E+03	2.10E+02	9.22E-03	3.50E-04	2.79E-06	2.10E-07	3.07
GlyRβ_DIM_008_10_2P	FSIVGSLPRDOOJ	4.06E+03	3.30E+02	1.03E-02	5.00E-04	2.53E-06	2.40E-07	2.37
GlyRβ_DIM_008_10_1P	FSIVGSLPRDOJ	4.48E+03	3.60E+02	1.12E-02	5.00E-04	2.49E-06	2.30E-07	2.31
GlyRβ_DIM_008_10_0P	FSIVGSLPRDJ	0.00E+00	0.00E+00	0.00E+00	0.00E+00	0.00E+00	0.00E+00	0.00E+00
GlyRβ_DIM_008_9_3P	FSIVGSLPROOOJ	3.11E+03	2.90E+02	1.25E-02	6.00E-04	4.03E-06	4.30E-07	2.17
GlyRβ_DIM_008_9_2P	FSIVGSLPROOJ	3.97E+03	3.80E+02	1.23E-02	6.00E-04	3.11E-06	3.40E-07	2.04
GlyRβ_DIM_008_9_1P	FSIVGSLPROJ	3.38E+03	1.49E+03	1.61E-02	7.00E-04	4.76E-06	3.10E-07	1.31
GlyRβ_DIM_008_9_0P	FSIVGSLPRJ	0.00E+00	0.00E+00	0.00E+00	0.00E+00	0.00E+00	0.00E+00	0.00E+00
GlyRβ_DIM_008_8_3P	FSIVGSLPOOOJ	1.48E+03	2.10E+02	1.26E-02	8.00E-04	8.46E-06	1.31E-06	2.01
GlyRβ_DIM_008_8_2P	FSIVGSLPOOJ	3.08E+03	2.80E+02	1.01E-02	5.00E-04	3.29E-06	3.50E-07	2.36
GlyRβ_DIM_008_8_1P	FSIVGSLPOJ	1.77E+03	7.40E+02	1.71E-02	1.40E-03	9.66E-06	6.40E-07	1.31
GlyRβ_DIM_008_8_0P	FSIVGSLPJ	0.00E+00	0.00E+00	0.00E+00	0.00E+00	0.00E+00	0.00E+00	0.00E+00
GlyRβ_DIM_008_7_3P	FSIVGSLOOOJ	2.16E+03	2.50E+02	1.08E-02	7.00E-04	4.99E-06	6.50E-07	1.39
GlyRβ_DIM_008_7_2P	FSIVGSLOOJ	0.00E+00	0.00E+00	0.00E+00	0.00E+00	0.00E+00	0.00E+00	0.00E+00
GlyRβ_DIM_008_7_1P	FSIVGSLOJ	0.00E+00	0.00E+00	0.00E+00	0.00E+00	0.00E+00	0.00E+00	0.00E+00
GlyRβ_DIM_008_7_0P	FSIVGSLJ	0.00E+00	0.00E+00	0.00E+00	0.00E+00	0.00E+00	0.00E+00	0.00E+00
GlyRβ_DIM_008_6_3P	FSIVGSOOOJ	2.66E+03	6.10E+02	1.74E-02	1.70E-03	6.53E-06	1.64E-06	1.3
GlyRβ_DIM_008_6_2P	FSIVGSOOJ	2.13E+03	6.70E+02	1.89E-02	2.30E-03	8.89E-06	3.02E-06	1.1
GlyRβ_DIM_008_6_1P	FSIVGSOJ	0.00E+00	0.00E+00	0.00E+00	0.00E+00	0.00E+00	0.00E+00	0.00E+00
GlyRβ_DIM_008_6_0P	FSIVGSJ	0.00E+00	0.00E+00	0.00E+00	0.00E+00	0.00E+00	0.00E+00	0.00E+00
GlyRβ_DIM_008_5_3P	FSIVGOOOJ	0.00E+00	0.00E+00	0.00E+00	0.00E+00	0.00E+00	0.00E+00	0.00E+00
GlyRβ_DIM_008_5_2P	FSIVGOOJ	1.63E+03	4.80E+02	1.92E-02	1.90E-03	1.18E-05	3.70E-06	1.2
GlyRβ_DIM_008_5_1P	FSIVGOJ	0.00E+00	0.00E+00	0.00E+00	0.00E+00	0.00E+00	0.00E+00	0.00E+00
GlyRβ_DIM_008_5_0P	FSIVGJ	0.00E+00	0.00E+00	0.00E+00	0.00E+00	0.00E+00	0.00E+00	0.00E+00
GlyRβ_DIM_008_4_3P	FSIVOOOJ	0.00E+00	0.00E+00	0.00E+00	0.00E+00	0.00E+00	0.00E+00	0.00E+00
GlyRβ_DIM_008_4_2P	FSIVOOJ	0.00E+00	0.00E+00	0.00E+00	0.00E+00	0.00E+00	0.00E+00	0.00E+00

GlyRβ_DIM_008_4_1P	FSIVOJ	2.88E+03	5.60E+02	1.41E-02	1.60E-03	1.41E-05	1.60E-06	1.1
GlyRβ_DIM_008_4_0P	FSIVJ	0.00E+00	0.00E+00	0.00E+00	0.00E+00	0.00E+00	0.00E+00	0.00E+00
GlyRβ_DIM_008_10_3P_Ala001	**A**SIVGSLPRDOOOJ	0.00E+00	0.00E+00	0.00E+00	0.00E+00	0.00E+00	0.00E+00	0.00E+00
GlyRβ_DIM_008_10_3P_Ala002	F**A**IVGSLPRDOOOJ	0.00E+00	0.00E+00	0.00E+00	0.00E+00	0.00E+00	0.00E+00	0.00E+00
GlyRβ_DIM_008_10_3P_Ala003	FS**A**VGSLPRDOOOJ	0.00E+00	0.00E+00	0.00E+00	0.00E+00	0.00E+00	0.00E+00	0.00E+00
GlyRβ_DIM_008_10_3P_Ala004	FSI**A**GSLPRDOOOJ	0.00E+00	0.00E+00	0.00E+00	0.00E+00	0.00E+00	0.00E+00	0.00E+00
GlyRβ_DIM_008_10_3P_Ala005	FSIV**A**SLPRDOOOJ	1.45E+03	6.20E+02	2.77E-02	3.00E-03	1.91E-05	8.40E-06	0.892
GlyRβ_DIM_008_10_3P_Ala006	FSIVG**A**LPRDOOOJ	1.62E+03	8.20E+02	2.86E-02	3.80E-03	1.76E-05	9.20E-06	1.42
GlyRβ_DIM_008_10_3P_Ala007	FSIVGS**A**PRDOOOJ	1.30E+03	5.90E+02	2.99E-02	3.00E-03	2.29E-05	1.06E-05	1.3
GlyRβ_DIM_008_10_3P_Ala008	FSIVGSL**A**RDOOOJ	2.59E+03	2.43E+03	4.80E-02	7.60E-03	1.85E-05	1.06E-05	1.2
GlyRβ_DIM_008_10_3P_FP001	**Y**SIVGSLPRDOOOJ	1.72E+03	5.00E+01	2.19E-03	4.00E-05	1.27E-06	4.00E-08	7.53
GlyRβ_DIM_008_10_3P_FP002	**W**SIVGSLPRDOOOJ	2.05E+03	8.00E+01	3.62E-03	9.00E-05	1.76E-06	8.00E-08	4.18
GlyRβ_DIM_008_10_3P_FP003	F**G**IVGSLPRDOOOJ	0.00E+00	0.00E+00	0.00E+00	0.00E+00	0.00E+00	0.00E+00	0.00E+00
GlyRβ_DIM_008_10_3P_FP004	F**T**IVGSLPRDOOOJ	9.20E+03	1.43E+03	1.59E-02	1.10E-03	1.73E-06	2.90E-07	1.39
GlyRβ_DIM_008_10_3P_FP005	F**W**IVGSLPRDOOOJ	0.00E+00	0.00E+00	0.00E+00	0.00E+00	0.00E+00	0.00E+00	0.00E+00
GlyRβ_DIM_008_10_3P_FP006	FS**L**VGSLPRDOOOJ	2.87E+03	3.00E+02	1.43E-02	7.00E-04	5.00E-06	5.80E-07	2.32
GlyRβ_DIM_008_10_3P_FP007	FS**V**VGSLPRDOOOJ	5.15E+03	7.60E+02	1.67E-02	1.20E-03	3.24E-06	5.30E-07	1.27
GlyRβ_DIM_008_10_3P_FP008	FS**M**VGSLPRDOOOJ	3.65E+03	3.90E+02	1.37E-02	8.00E-04	3.74E-06	4.50E-07	1.97
GlyRβ_DIM_008_10_3P_FP009	FSI**I**GSLPRDOOOJ	3.84E+03	3.70E+02	1.29E-02	7.00E-04	3.37E-06	3.60E-07	2.44
GlyRβ_DIM_008_10_3P_FP010	FSI**W**GSLPRDOOOJ	0.00E+00	0.00E+00	0.00E+00	0.00E+00	0.00E+00	0.00E+00	0.00E+00
GlyRß_QUAD_008_4-0_1	FSIVGSLPOOOOJJ	3.23E+04	7.00E+02	1.43E-02	2.00E-04	4.43E-07	1.20E-08	31
GlyRß_QUAD_008_3-1_1	FSIVGSLPOOOJOJ	2.81E+04	8.00E+02	1.36E-02	3.00E-04	4.84E-07	1.70E-08	23
GlyRß_QUAD_008_2-2_1	FSIVGSLPOOJOOJ	2.63E+04	8.00E+02	1.37E-02	3.00E-04	5.21E-07	1.80E-08	22.8
GlyRß_QUAD_008_1-3_1	FSIVGSLPOJOOOJ	2.72E+04	1.10E+03	1.72E-02	4.00E-04	6.32E-07	2.80E-08	18.8
GlyRß_QUAD_008_0-4_1	FSIVGSLPJOOOOJ	2.14E+04	8.00E+02	1.59E-02	3.00E-04	7.43E-07	3.10E-08	21.5
GlyRß_QUAD_008_4-0_2	FSIVGSLPOOOOJJ	1.90E+04	5.00E+02	1.29E-02	3.00E-04	6.79E-07	2.30E-08	37.5
GlyRß_QUAD_008_3-1_2	FSIVGSLPOOOJOJ	1.86E+04	5.00E+02	1.27E-02	2.00E-04	6.83E-07	2.40E-08	30.8
GlyRß_QUAD_008_2-2_2	FSIVGSLPOOJOOJ	1.83E+04	6.00E+02	1.26E-02	3.00E-04	6.89E-07	2.70E-08	27.7
GlyRß_QUAD_008_1-3_2	FSIVGSLPOJOOOJ	2.07E+04	9.00E+02	1.61E-02	4.00E-04	7.78E-07	3.90E-08	21.1
GlyRß_QUAD_008_0-4_2	FSIVGSLPJOOOOJ	1.71E+04	6.00E+02	1.46E-02	3.00E-04	8.54E-07	3.50E-08	23.6
GlyRß_QUAD_008_4-0_3	FSIVGSLPOOOOJJ	1.96E+04	6.00E+02	1.36E-02	3.00E-04	6.94E-07	2.70E-08	40.2
GlyRß_QUAD_008_3-1_3	FSIVGSLPOOOJOJ	1.85E+04	6.00E+02	1.27E-02	3.00E-04	6.86E-07	3.10E-08	28
GlyRß_QUAD_008_2-2_3	FSIVGSLPOOJOOJ	1.72E+04	6.00E+02	1.25E-02	3.00E-04	7.27E-07	3.10E-08	25.2
GlyRß_QUAD_008_1-3_3	FSIVGSLPOJOOOJ	1.82E+04	8.00E+02	1.51E-02	3.00E-04	8.30E-07	3.90E-08	20.1
GlyRß_QUAD_008_0-4_3	FSIVGSLPJOOOOJ	1.49E+04	6.00E+02	1.42E-02	3.00E-04	9.53E-07	4.60E-08	21.8
GlyRß_QUAD_007_4-0	FSIVGSLOOOOJJ	1.78E+04	2.90E+03	2.02E-02	1.30E-03	1.13E-06	2.00E-07	9.3
GlyRß_QUAD_007_3-1	FSIVGSLOOOJOJ	1.74E+04	3.20E+03	2.38E-02	1.50E-03	1.37E-06	2.60E-07	7.54
GlyRß_QUAD_007_2-2	FSIVGSLOOJOOJ	6.78E+03	6.10E+02	1.14E-02	4.00E-04	1.68E-06	1.60E-07	17
GlyRß_QUAD_007_1-3	FSIVGSLOJOOOJ	9.18E+03	7.70E+02	1.01E-02	4.00E-04	1.10E-06	1.00E-07	12.1
GlyRß_QUAD_007_0-4	FSIVGSLJOOOOJ	1.47E+04	2.20E+03	1.06E-02	1.10E-03	7.21E-07	1.29E-07	5.55
GlyRß_QUAD_006_4-0	FSIVGSOOOOJJ	2.03E+04	3.10E+03	1.49E-02	1.10E-03	7.34E-07	1.25E-07	7.09
GlyRß_QUAD_006_3-1	FSIVGSOOOJOJ	8.93E+03	1.43E+03	9.09E-03	8.10E-04	1.02E-06	1.90E-07	7.91
GlyRß_QUAD_006_2-2	FSIVGSOOJOOJ	2.35E+04	3.50E+03	1.23E-02	1.30E-03	5.23E-07	9.60E-08	4.77

GlyRß_QUAD_006_1-3	FSIVGSOJOOOJ	7.55E+03	7.90E+02	1.02E-02	5.00E-04	1.35E-06	1.60E-07	11.9
GlyRß_QUAD_006_0-4	FSIVGSJOOOOJ	2.14E+04	1.80E+03	7.64E-03	6.30E-04	3.57E-07	4.20E-08	7.39
GlyRß_QUAD_005_4-0	FSIVGOOOOJJ	1.78E+03	2.42E+03	1.98E-03	1.69E-03	1.11E-06	1.79E-06	4.91
GlyRß_QUAD_005_3-1	FSIVGOOOJOJ	1.07E+04	3.80E+03	1.78E-03	2.05E-03	1.66E-07	1.99E-07	2.83
GlyRß_QUAD_005_2-2	FSIVGOOJOOJ	6.55E+03	5.81E+03	1.98E-03	2.83E-03	3.02E-07	5.09E-07	2.05
GlyRß_QUAD_005_1-3	FSIVGOJOOOJ	7.43E+03	4.99E+03	1.42E-03	3.21E-03	1.91E-07	4.50E-07	1.67
GlyRß_QUAD_005_0-4	FSIVGJOOOOJ	8.20E+03	1.78E+03	3.77E-03	1.27E-03	4.60E-07	1.84E-07	4.04
GlyRß_OCT_008_4-0-0	FSIVGSLPOOOOJJJ	1.03E+05	6.00E+03	9.21E-03	2.00E-04	8.94E-08	5.30E-09	11.4
GlyRß_OCT_008_3-0-1	FSIVGSLPOOOJJOJ	7.35E+04	4.40E+03	7.70E-03	1.70E-04	1.05E-07	7.00E-09	7.6
GlyRß_OCT_008_3-1-0	FSIVGSLPOOOJOJJ	4.69E+04	2.90E+03	7.45E-03	2.00E-04	1.59E-07	1.10E-08	6.47
GlyRß_OCT_008_2-0-2	FSIVGSLPOOJJOOJ	4.49E+04	2.40E+03	8.04E-03	1.90E-04	1.79E-07	1.00E-08	8.03
GlyRß_OCT_008_2-1-1	FSIVGSLPOOJOJOJ	3.83E+04	2.40E+03	8.19E-03	2.20E-04	2.14E-07	1.40E-08	6.41
GlyRß_OCT_008_2-2-0	FSIVGSLPOOJOOJJ	3.67E+04	9.00E+02	6.75E-03	1.30E-04	1.84E-07	6.00E-09	19.4
GlyRß_OCT_008_1-0-3	FSIVGSLPOJJOOOJ	2.80E+04	8.00E+02	6.11E-03	1.20E-04	2.18E-07	8.00E-09	14.4
GlyRß_OCT_008_1-1-2	FSIVGSLPOJOJOOJ	3.32E+04	1.30E+03	6.92E-03	1.50E-04	2.08E-07	9.00E-09	10.9
GlyRß_OCT_008_1-2-1	FSIVGSLPOJOOJOJ	3.84E+04	2.00E+03	7.71E-03	1.90E-04	2.01E-07	1.20E-08	7.52
GlyRß_OCT_008_1-3-0	FSIVGSLPOJOOOJJ	3.34E+04	1.90E+03	8.04E-03	2.00E-04	2.41E-07	1.50E-08	6.88
GlyRß_OCT_008_0-0-4	FSIVGSLPJJOOOOJ	2.67E+04	6.00E+02	6.55E-03	1.00E-04	2.45E-07	7.00E-09	20.1
GlyRß_OCT_008_0-1-3	FSIVGSLPJOJOOOJ	3.07E+04	9.00E+02	6.10E-03	1.30E-04	1.99E-07	7.00E-09	15.4
GlyRß_OCT_008_0-2-2	FSIVGSLPJOOJOOJ	3.21E+04	1.10E+03	6.44E-03	1.30E-04	2.01E-07	8.00E-09	11.6
GlyRß_OCT_008_0-3-1	FSIVGSLPJOOOJOJ	3.48E+04	1.30E+03	6.71E-03	1.30E-04	1.93E-07	8.00E-09	10
GlyRß_OCT_008_0-4-0	FSIVGSLPJOOOOJJ	3.40E+04	1.50E+03	7.27E-03	1.60E-04	2.14E-07	1.10E-08	8.32

Supplementary Table 2: Observed and predicted dimeric, tetrameric and octameric peptides binding rates. The observed on-, off-rate, and K_D value and corresponding predictions for each peptide sequence are given. We used the median of duplicated sequences, i.e., repeated measurements.

Peptide	Observed K_{on} [$M^{-1}s^{-1}$]	Predicted K_{on} [$M^{-1}s^{-1}$]	Observed $log(K_{off})^2$ [s^{-1}]	Predicted $log(K_{off})^2$ [s^{-1}]	Observed $log(K_D)^2$ [M]	Predicted $log(K_D)^2$ [M]
GlyRβ_DIM_008_8_3P_WT001	1.63E+03	2.84E+03	3.23E+01	1.86E+01	1.75E+02	1.51E+02
GlyRβ_DIM_002	3.59E+03	7.08E+03	2.03E+01	1.52E+01	1.61E+02	1.56E+02
GlyRβ_DIM_005*	1.02E+04	4.76E+03	1.22E+01	1.81E+01	1.62E+02	1.59E+02
GlyRβ_DIM_006	6.35E+03	6.06E+03	1.54E+01	1.81E+01	1.61E+02	1.61E+02
GlyRβ_DIM_007	5.15E+03	2.74E+03	1.83E+01	2.05E+01	1.64E+02	1.57E+02
GlyRβ_DIM_008	2.67E+03	4.67E+03	2.15E+01	1.84E+01	1.55E+02	1.64E+02
GlyRβ_DIM_008_10_2P	4.06E+03	4.06E+03	2.09E+01	1.98E+01	1.66E+02	1.61E+02
GlyRβ_DIM_008_10_1P	4.48E+03	3.66E+03	2.02E+01	1.87E+01	1.66E+02	1.53E+02
GlyRβ_DIM_008_9_3P	3.11E+03	3.54E+03	1.92E+01	1.93E+01	1.54E+02	1.58E+02
GlyRβ_DIM_008_9_2P	3.97E+03	3.42E+03	1.93E+01	1.93E+01	1.61E+02	1.58E+02
GlyRβ_DIM_008_9_1P	3.38E+03	3.70E+03	1.70E+01	1.93E+01	1.50E+02	1.55E+02
GlyRβ_DIM_008_8_2P	3.08E+03	1.93E+03	2.11E+01	2.19E+01	1.59E+02	1.53E+02
GlyRβ_DIM_008_8_1P	1.77E+03	3.38E+03	1.66E+01	2.27E+01	1.33E+02	1.68E+02
GlyRβ_DIM_008_7_3P	2.16E+03	2.54E+03	2.05E+01	1.71E+01	1.49E+02	1.63E+02
GlyRβ_DIM_008_6_3P	2.66E+03	3.59E+03	1.64E+01	2.05E+01	1.43E+02	1.42E+02
GlyRβ_DIM_008_6_2P	2.13E+03	4.25E+03	1.57E+01	1.87E+01	1.35E+02	1.42E+02
GlyRβ_DIM_008_5_2P	1.63E+03	3.06E+03	1.56E+01	3.91E+01	1.29E+02	1.42E+02
GlyRβ_DIM_008_4_1P	2.88E+03	3.28E+03	1.82E+01	2.38E+01	1.25E+02	1.45E+02
GlyRβ_DIM_008_10_3P_Ala005	1.45E+03	2.33E+03	1.29E+01	1.22E+01	1.18E+02	1.31E+02
GlyRβ_DIM_008_10_3P_Ala006	1.62E+03	4.71E+03	1.26E+01	1.22E+01	1.20E+02	1.27E+02
GlyRβ_DIM_008_10_3P_Ala007	1.30E+03	2.14E+03	1.23E+01	1.28E+01	1.14E+02	1.26E+02
GlyRβ_DIM_008_10_3P_Ala008	2.59E+03	2.15E+03	9.22E+00	1.31E+01	1.19E+02	1.24E+02
GlyRβ_DIM_008_10_3P_FP001	1.72E+03	3.15E+03	3.75E+01	2.08E+01	1.84E+02	1.65E+02
GlyRβ_DIM_008_10_3P_FP002	2.05E+03	3.27E+03	3.16E+01	2.46E+01	1.76E+02	1.70E+02
GlyRβ_DIM_008_10_3P_FP004	9.20E+03	3.44E+03	1.72E+01	1.98E+01	1.76E+02	1.59E+02
GlyRβ_DIM_008_10_3P_FP006	2.87E+03	4.07E+03	1.80E+01	1.88E+01	1.49E+02	1.59E+02
GlyRβ_DIM_008_10_3P_FP007	5.15E+03	3.46E+03	1.67E+01	2.04E+01	1.60E+02	1.55E+02
GlyRβ_DIM_008_10_3P_FP008	3.65E+03	4.11E+03	1.84E+01	1.90E+01	1.56E+02	1.61E+02
GlyRβ_DIM_008_10_3P_FP009	3.84E+03	3.51E+03	1.89E+01	2.12E+01	1.59E+02	1.59E+02
GlyRß_QUAD_008_4-0_1	1.96E+04	1.55E+04	1.85E+01	1.92E+01	2.02E+02	1.97E+02

GlyRß_QUAD_008_3-1_1	1.86E+04	1.58E+04	1.91E+01	2.32E+01	2.02E+02	1.99E+02
GlyRß_QUAD_008_2-2_1	1.83E+04	1.84E+04	1.91E+01	1.90E+01	2.01E+02	2.01E+02
GlyRß_QUAD_008_1-3_1	2.07E+04	1.26E+04	1.70E+01	1.87E+01	1.98E+02	1.99E+02
GlyRß_QUAD_008_0-4_1	1.71E+04	1.88E+04	1.79E+01	2.00E+01	1.95E+02	2.06E+02
GlyRß_QUAD_007_4-0	1.78E+04	1.14E+04	1.52E+01	1.97E+01	1.88E+02	1.85E+02
GlyRß_QUAD_007_3-1	1.74E+04	1.09E+04	1.40E+01	2.13E+01	1.82E+02	1.91E+02
GlyRß_QUAD_007_2-2	6.78E+03	1.69E+04	2.00E+01	1.89E+01	1.77E+02	1.96E+02
GlyRß_QUAD_007_1-3	9.18E+03	1.29E+04	2.11E+01	1.79E+01	1.88E+02	1.86E+02
GlyRß_QUAD_007_0-4	1.47E+04	1.44E+04	2.07E+01	2.05E+01	2.00E+02	1.92E+02
GlyRß_QUAD_006_4-0	2.03E+04	1.57E+04	1.77E+01	1.81E+01	2.00E+02	1.94E+02
GlyRß_QUAD_006_3-1	8.93E+03	1.94E+04	2.21E+01	1.71E+01	1.90E+02	2.08E+02
GlyRß_QUAD_006_2-2	2.35E+04	1.19E+04	1.93E+01	2.05E+01	2.09E+02	1.92E+02
GlyRß_QUAD_006_1-3	7.55E+03	1.71E+04	2.10E+01	2.06E+01	1.83E+02	2.10E+02
GlyRß_QUAD_006_0-4	2.14E+04	1.55E+04	2.38E+01	2.04E+01	2.20E+02	1.98E+02
GlyRß_QUAD_005_4-0	1.78E+03	1.07E+04	3.87E+01	3.12E+01	1.88E+02	2.26E+02
GlyRß_QUAD_005_3-1	1.07E+04	6.83E+03	4.01E+01	3.32E+01	2.44E+02	2.09E+02
GlyRß_QUAD_005_2-2	6.55E+03	9.61E+03	3.87E+01	3.05E+01	2.25E+02	2.29E+02
GlyRß_QUAD_005_1-3	7.43E+03	7.50E+03	4.30E+01	3.75E+01	2.39E+02	2.12E+02
GlyRß_QUAD_005_0-4	8.20E+03	9.36E+03	3.11E+01	3.92E+01	2.13E+02	2.28E+02
GlyRß_OCT_008_4-0-0	1.03E+05	4.10E+04	2.20E+01	2.36E+01	2.63E+02	2.37E+02
GlyRß_OCT_008_3-0-1	7.35E+04	4.73E+04	2.37E+01	2.47E+01	2.58E+02	2.27E+02
GlyRß_OCT_008_3-1-0	4.69E+04	4.39E+04	2.40E+01	2.41E+01	2.45E+02	2.38E+02
GlyRß_OCT_008_2-0-2	4.49E+04	3.66E+04	2.33E+01	2.52E+01	2.41E+02	2.37E+02
GlyRß_OCT_008_2-1-1	3.83E+04	5.65E+04	2.31E+01	2.42E+01	2.36E+02	2.47E+02
GlyRß_OCT_008_2-2-0	3.67E+04	4.40E+04	2.50E+01	2.33E+01	2.41E+02	2.40E+02
GlyRß_OCT_008_1-0-3	2.80E+04	3.41E+04	2.60E+01	2.40E+01	2.35E+02	2.35E+02
GlyRß_OCT_008_1-1-2	3.32E+04	4.03E+04	2.47E+01	2.43E+01	2.37E+02	2.36E+02
GlyRß_OCT_008_1-2-1	3.84E+04	5.29E+04	2.37E+01	2.36E+01	2.38E+02	2.45E+02
GlyRß_OCT_008_1-3-0	3.34E+04	3.92E+04	2.33E+01	2.42E+01	2.32E+02	2.38E+02
GlyRß_OCT_008_0-0-4	2.67E+04	3.18E+04	2.53E+01	2.53E+01	2.32E+02	2.36E+02
GlyRß_OCT_008_0-1-3	3.07E+04	3.32E+04	2.60E+01	2.50E+01	2.38E+02	2.37E+02
GlyRß_OCT_008_0-2-2	3.21E+04	4.22E+04	2.55E+01	2.49E+01	2.38E+02	2.40E+02

GlyRß_OCT_008_0-3-1	3.48E+04	5.85E+04	2.50E+01	2.40E+01	2.39E+02	2.45E+02
GlyRß_OCT_008_0-4-0	3.40E+04	3.74E+04	2.42E+01	2.46E+01	2.36E+02	2.41E+02

Supplementary Table 3: Purity of preparatively synthesized and purified peptides used for FPS, BLI, ITC, TRIC (Figure 2) and microarray-based (Figure 4 G, H) measurements.

Peptide Sequence	Code	M.W. [Da]	Purity [%]
FSIVGSLPOOJ	e=8, o=2	2328.73	90.8
FSIVGSLPOJ	e=8, o=1	2038.42	89.8
FSIVGOOJ	e=5, o=2	1734.02	>98
FSIVGOJ	e=5, o=1	1443.71	96
FSIVGSLPOOOOJJ	e=8, o_1=4, o_2=0	5928.89	>90
FSIVGSLPOOJOOJ	e=8, o_1=2, o_2=2	5348.26	92.7
FSIVGSLPJOOOOJ	e=8, o_1=0, o_2=4	4767.62	84

Supplementary Table 4: Purity of crude peptides synthesized in 2 µmol scale for high-throughput FPS measurements. Multimeric peptides used for FPS and BLI measurements (Figure 3) were analysed by LC-MS.

Peptide Sequence	Code	M.W. [Da]	Purity [%]
FNFSGYGMGHOOJ	N.D.	2922.24	72.4
ANFSGYGMGHOOJ	N.D.	2770.05	79.5
FNASGYGMGHOOJ	N.D.	2770.05	69.6
FSIVGSLPOOOJOJ	e=8, o_1=3, o_2=1	5638.57	52.2
FSIVGSLPOOJOOJ	e=8, o_1=2, o_2=2	5348.26	54.6
FSIVGSLPOJOOOJ	e=8, o_1=1, o_2=3	5057.94	57.6
FSIVGSLPJOOOOJ	e=8, o_1=0, o_2=4	4767.62	60.7
FSIVGSLPOOOOJJJ	e=8, o_1=4, o_2=0, o_3=0	12095.13	N.D.
FSIVGSLPOOJJOOJ	e=8, o_1=2, o_2=0, o_3=2	10225.06	N.D.
FSIVGSLPOOJOOJJ	e=8, o_1=2, o_2=2, o_3=0	10805.69	N.D.
FSIVGSLPOJJOOOJ	e=8, o_1=1, o_2=0, o_3=3	9354.11	N.D.

Supplementary References

KIM, E. Y., SCHRADER, N., SMOLINSKY, B., BEDET, C., VANNIER, C., SCHWARZ, G. & SCHINDELIN, H. 2006. Deciphering the structural framework of glycine receptor anchoring by gephyrin. *The EMBO journal,* 25**,** 1385-95.
