# Supplementary material for: Multivalent binding kinetics resolved by fluorescence proximity sensing
Source: Commun Biol. 2022 Oct 7;5:1070. doi: 10.1038/s42003-022-03997-3 (PMC9546861; doi:10.1038/s42003-022-03997-3)
Supplement: Supplementary file 4 — Reporting Summary [file 42003_2022_3997_MOESM4_ESM.pdf]

nature portfolio

Corresponding author(s): Hans Michael Maric

Last updated by author(s): Aug 31, 2022

Reporting Summary

Nature Portfolio wishes to improve the reproducibility of the work that we publish. This form provides structure for consistency and transparency in reporting. For further information on Nature Portfolio policies, see our Editorial Policies and the Editorial Policy Checklist.

Statistics

For all statistical analyses, confirm that the following items are present in the figure legend, table legend, main text, or Methods section.

n/a	Confirmed	
☐	☒	The exact sample size (*n*) for each experimental group/condition, given as a discrete number and unit of measurement
☒	☐	A statement on whether measurements were taken from distinct samples or whether the same sample was measured repeatedly
☒	☐	The statistical test(s) used AND whether they are one- or two-sided<br>*Only common tests should be described solely by name; describe more complex techniques in the Methods section.*
☒	☐	A description of all covariates tested
☒	☐	A description of any assumptions or corrections, such as tests of normality and adjustment for multiple comparisons
☐	☒	A full description of the statistical parameters including central tendency (e.g. means) or other basic estimates (e.g. regression coefficient) AND variation (e.g. standard deviation) or associated estimates of uncertainty (e.g. confidence intervals)
☒	☐	For null hypothesis testing, the test statistic (e.g. *F*, *t*, *r*) with confidence intervals, effect sizes, degrees of freedom and *P* value noted<br>*Give P values as exact values whenever suitable.*
☒	☐	For Bayesian analysis, information on the choice of priors and Markov chain Monte Carlo settings
☒	☐	For hierarchical and complex designs, identification of the appropriate level for tests and full reporting of outcomes
☐	☒	Estimates of effect sizes (e.g. Cohen's *d*, Pearson's *r*), indicating how they were calculated

Our web collection on statistics for biologists contains articles on many of the points above.

Software and code

Policy information about availability of computer code

Data collection	N.A.
Data analysis	https://github.com/spaenigs/fluorescence-proximity-sensing

For manuscripts utilizing custom algorithms or software that are central to the research but not yet described in published literature, software must be made available to editors and reviewers. We strongly encourage code deposition in a community repository (e.g. GitHub). See the Nature Portfolio guidelines for submitting code & software for further information.

Data

Policy information about availability of data

All manuscripts must include a data availability statement. This statement should provide the following information, where applicable:

- Accession codes, unique identifiers, or web links for publicly available datasets
- A description of any restrictions on data availability
- For clinical datasets or third party data, please ensure that the statement adheres to our policy

All data generated or analysed during this study are included in this published article (and its supplementary information files).

Human research participants

Policy information about studies involving human research participants and Sex and Gender in Research.

Reporting on sex and gender	Does not apply.
Population characteristics	Does not apply.
Recruitment	Does not apply.
Ethics oversight	Does not apply.

Note that full information on the approval of the study protocol must also be provided in the manuscript.

Field-specific reporting

Please select the one below that is the best fit for your research. If you are not sure, read the appropriate sections before making your selection.

☒ Life sciences ☐ Behavioural & social sciences ☐ Ecological, evolutionary & environmental sciences

For a reference copy of the document with all sections, see nature.com/documents/nr-reporting-summary-flat.pdf

Life sciences study design

All studies must disclose on these points even when the disclosure is negative.

Sample size	Sample sizes of the biophysical measurements in this manuscript are in agreement with leading examples in the literature.
Data exclusions	Exclusion of datapoints in biophysical measurements was done based on the algorithms included in the proprietary softwares used for instrument control (specifically relating to supplementary figure 9 in which TRIC measurements are shown).
Replication	The technical replicates of the biophysical measurements shown were analyzed in terms of their standard deviation.
Randomization	Does not apply.
Blinding	Does not apply.

Reporting for specific materials, systems and methods

We require information from authors about some types of materials, experimental systems and methods used in many studies. Here, indicate whether each material, system or method listed is relevant to your study. If you are not sure if a list item applies to your research, read the appropriate section before selecting a response.

Materials & experimental systems

n/a	Involved in the study	
☐	☒	Antibodies
☒	☐	Eukaryotic cell lines
☒	☐	Palaeontology and archaeology
☒	☐	Animals and other organisms
☒	☐	Clinical data
☒	☐	Dual use research of concern

Methods

n/a	Involved in the study	
☒	☐	ChIP-seq
☒	☐	Flow cytometry
☒	☐	MRI-based neuroimaging

Antibodies

Antibodies used	1) Mouse monoclonal Anti-Gephyrin antibody (clone: 3B11) - Synaptic Systems 2) Goat Anti-Mouse IgG (H+L) secondary antibody HRP conjugated (catalog no.: 31430) - Invitrogen
Validation	Validation statement on the manufacturers website: https://sysy.com/product/147111<br>Our group used the primary anti-gephyrin antibody successfully in the past for microarray-based binding assays as shown in this study (https://pubmed.ncbi.nlm.nih.gov/33364586/)
